# Immune checkpoint inhibitors in osteosarcoma: A hopeful and challenging future

**DOI:** 10.3389/fphar.2022.1031527

**Published:** 2022-10-17

**Authors:** Zeng Zhang, Xin Tan, Zengxin Jiang, Hao Wang, Hengfeng Yuan

**Affiliations:** ^1^ Department of Orthopedics, Shanghai Sixth People’s Hospital, Shanghai Jiaotong University, Shanghai, China; ^2^ Department of Orthopedics, The Second Affiliated Hospital of Chongqing Medical University, Chongqing, China

**Keywords:** osteosarcoma, immunotherapy, immune checkpoint inhibitor, side effects, mechanism

## Abstract

Osteosarcoma (OS), the most common malignant tumor in the musculoskeletal system, mainly occurs in adolescents. OS results in high mortality and disability rates due to a fatal metastatic tendency and subsequent iatrogenic damage caused by surgery, radiotherapy and chemotherapy. Recently, immunotherapies have resulted in promising prognoses with reduced side effects compared with traditional therapies. Immune checkpoint inhibitors (ICIs), which are a representative immunotherapy for OS, enhance the antitumor effects of immune cells. ICIs have shown satisfactory outcomes in other kinds of malignant tumors, especially hemopoietic tumors. However, there is still a high percentage of failures or severe side effects associated with the use of ICIs to treat OS, leading to far worse outcomes. To reveal the underlying mechanisms of drug resistance and side effects, recent studies elucidated several possible reasons, including the activation of other inhibitory immune cells, low immune cell infiltration in the tumor microenvironment, different immune properties of OS subtypes, and the involvement of osteogenesis and osteolysis. According to these mechanisms, researchers have developed new methods to overcome the shortcomings of ICIs. This review summarizes the recent breakthroughs in the use of ICIs to treat OS. Although numerous issues have not been solved yet, ICIs are still the most promising treatment options to cure OS in the long run.

## Introduction

Osteosarcoma (OS), one of the most common malignant bone tumors, tends to affect children and adolescents with a median age of 16 years (Siegel et al., 2018). OS mainly occurs in the long bones of the extremities, such as the tibia, femur and humerus. For nonmetastatic OS patients, a combination of traditional therapies, including wide resection, radiotherapy, and chemotherapy, leads to a 60%–70% 5-year survival rate (Anderson, 2016). Unfortunately, for metastatic patients, a high recurrence rate and low survival rate of nearly 20% make OS treatment challenging (Saraf et al., 2018; Wang et al., 2019).

Treating OS patients surgically with tumor-free resection is a traditional but effective method. According to the size and invasiveness of the tumor, typical operative plans include amputation, rotationplasty and limb-salvage surgery. Traditionally, radical resection of the primary tumor has a higher opportunity to thoroughly remove malignant tumor cells. Therefore, radical resection surgeries such as amputation and rotationplasty are supposed to result in higher survival rates, even though these treatments seriously worsen quality of life (Bläsius et al., 2022). However, recent studies have reached the opposite conclusion. Han et al. (2016) conducted a meta-analysis comparing the effect of limb-salvage surgery and amputation, and the results showed that limb-salvage surgery led to a comparable survival rate with much better quality of life. In addition to surgery, chemotherapy is another method to treat malignant tumors. Commonly used chemotherapeutic drugs for OS are methotrexate, doxorubicin, cisplatin, ifosfamide, and adriamycin (Piperno-Neumann et al., 2016). For localized nonmetastatic OS, tumor resection surgery combined with chemotherapy had a 5-year survival rate of approximately 60%–70%, while the survival rate for metastatic cases was approximately 20% (Senerchia et al., 2017; Ferrari et al., 2018). Tumor resection and adjuvant chemotherapy are the standard treatment for OS at present, but for metastatic patients with chemotherapeutic drug resistance, radiotherapy is another palliative option to extend patient lifespan. Unfortunately, for these palliative therapy patients, radiotherapy had an average survival of only approximately 6 months (Rahn et al., 2015). Radiotherapy can also be used as adjuvant therapy after resection surgery. Reports have shown that radiotherapy can decrease the possibility of local recurrence but does not increase the overall survival rate (Tinkle et al., 2019; Heng et al., 2020).

Immunotherapy has attracted attention from clinicians and researchers for its increased efficacy and reduced side effects (Dongye et al., 2022). Frequently used immunotherapies for OS include immune checkpoint inhibitors (ICIs), cytokines, adoptive T-cell therapy and cancer vaccines. These procedures can activate the restricted immune system in OS patients by targeting different kinds of immune cells (Lu et al., 2022). Among them, ICIs such as cytotoxic T-lymphocyte antigen 4 (CTLA-4) inhibitors, programmed cell death protein-1 (PD-1) and programmed death-ligand 1 (PD-L1) have shown great therapeutic effects on various kinds of malignant tumors with fewer side effects than traditional therapy, especially in treating melanoma and hematologic malignancies (Luke et al., 2022; Mei et al., 2022). However, for solid tumors such as OS, the prognosis of ICI intervention is not satisfactory (Meftahpour et al., 2022). This review introduces the outcomes of ICI interventions for OS and the related mechanisms, summarizes the current breakthroughs, and predicts the developmental direction of immunotherapies for OS in the future.

## Mechanisms of immune checkpoint inhibitors

**FIGURE 1:**
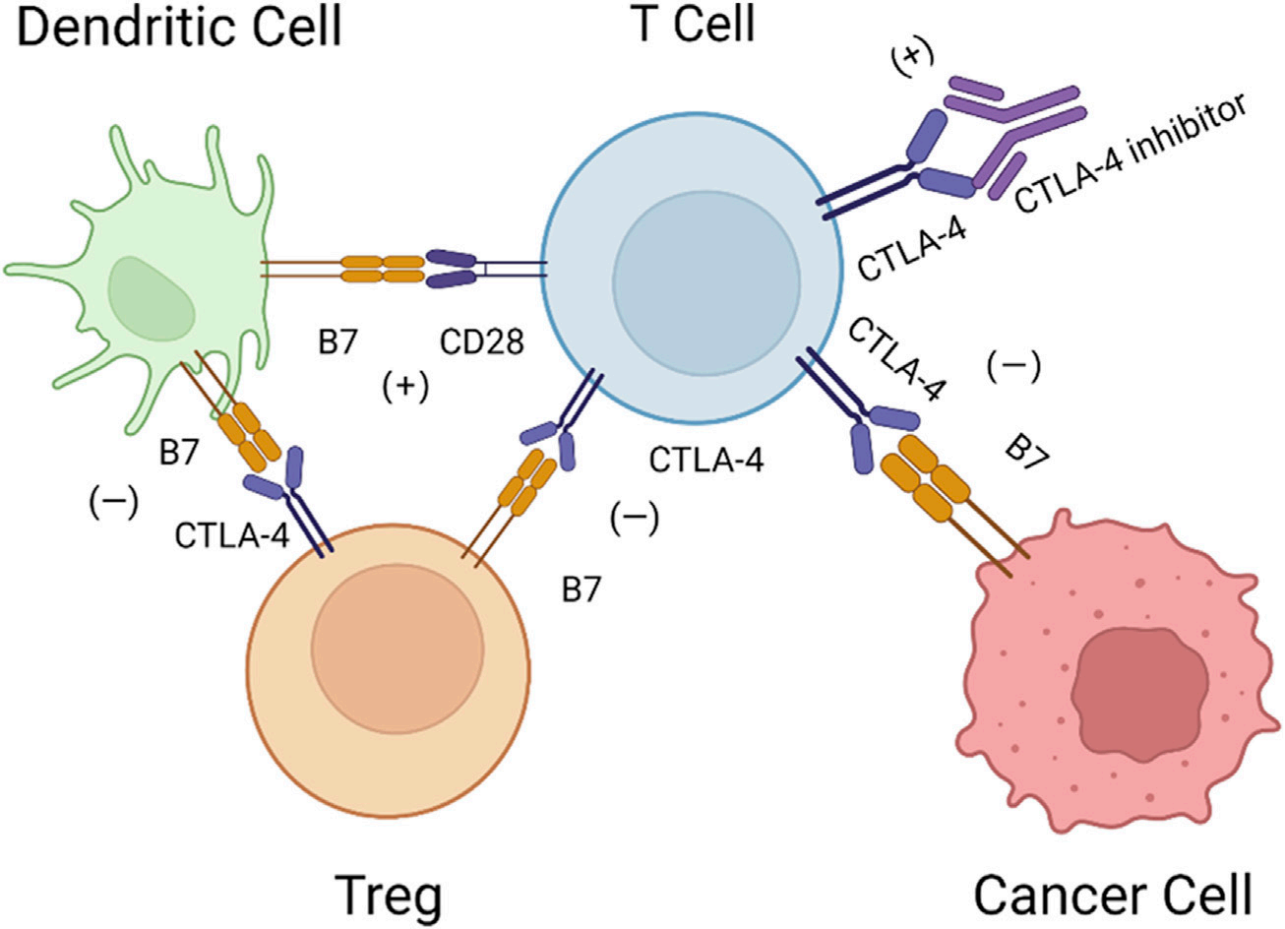
Mechanisms of CTLA-4. DCs activate T cells *via* B7-CD28, while CTLA-4 can bind with B7 on Tregs and cancer cells with higher affinity, and Tregs can inversely inhibit DCs *via* B7-CTLA-4. Therefore, CTLA-4 inhibitors may block this pathway to restore the activation of T cells.

CTLA-4, an immune checkpoint receptor protein that is highly expressed on the surface of T cells, plays a predominant role in inhibiting the functions of T cells (Lindsten et al., 1993). In detail, CTLA-4 is a type 1 transmembrane glycoprotein in the Ig superfamily that is composed of four domains: a signal peptide, an extracellular ligand-binding domain, a transmembrane domain, and a short cytoplasmic tail (Brunet et al., 1987; Ostrov et al., 2000). CTLA-4 and CD28 are both expressed on the surface of T cells and share the ligand B7, but they have opposite biological functions. Interactions between CD28 and the ligand B7 activate T cells and promote proliferation (Wang et al., 2016), while interactions between CTLA-4 and B7, including B7-1 (CD80) and B7-2 (CD86), have the opposite effects (Darlington et al., 2005). CTLA-4 has a higher affinity for B7 than CD28. Studies have suggested that CTLA-4 has an inhibitory effect on T cells through competitive binding to the ligand (Parry et al., 2005; Schneider et al., 2006). In addition, CTLA-4 can remove CD80 and CD86 on antigen-presenting cells (APCs) by preventing the binding of CD80 and CD86 with CD28 and trans-endocytosis, making T cells unable to accept immune signals (Qureshi et al., 2011). In Th-cell-specific CTLA-4 conditional-knockdown mice, CD80 and CD86 are highly expressed on APCs, indicating that CTLA-4 could inhibit the activation of T cells by restricting APCs. CTLA-4 could also reduce the activity of the transcription factors activator protein-1 (AP-1), nuclear factor of activated T cells (NFAT) and nuclear factor-κB (NF-κB), further decreasing the production of interleukin-2 (IL-2) (Fraser et al., 1999). IL-2 plays an important role in the interaction between CD28 and capZIP, a regulator of the actin cytoskeleton, interfering with the activation of T cells (Tian et al., 2015). In addition, CTLA-4 upregulates the activity of regulatory T cells (Tregs) and decreases helper T (Th) cells (Peggs et al., 2009). CTLA-4 is the direct target of forkhead box p3 (Foxp3), the linage-specifying transcription factor of Tregs (Marson et al., 2007). Tregs can reverse transmit signals to dendritic cells (DCs), inducing the expression of the tryptophan-catabolizing enzyme indoleamine 2,3-dioxygenase (IDO), consuming tryptophan and preventing the activation and proliferation of T cells (Fallarino et al., 2003). Recruitment of the serine/threonine phosphatase protein phosphatase 2A (PP2A) is mediated by CTLA-4 and inhibits the Akt signaling pathway, decreasing CD28-mediated glucose uptake by T cells and activating the PI3K pathway, promoting the proliferation of anergic T cells (Frauwirth et al., 2002; Intlekofer and Thompson, 2013). The detailed mechanisms of CTLA-4 are shown in Figure 1.

PD-1 is an immune checkpoint coinhibitory receptor protein that is highly expressed on the surface of T cells (Ishida et al., 1992). In a PD-1 defective animal model, researchers observed delayed-onset, organ-specific autoimmune diseases, including lupus-like syndrome and autoimmune-dilated cardiomyopathy, indicating the lymphocytes inhibit the functions of PD-1 (Nishimura et al., 1999; Okazaki et al., 2003). Similar to CTLA-4, PD-1 is also a type 1 transmembrane protein in the Ig superfamily. PD-1 has three parts: an extracellular N-terminal IgV-like domain, a transmembrane domain, and a cytoplasmic tail (Zhang et al., 2004). The immunoreceptor tyrosine-based inhibitory motif (ITIM) and immunoreceptor tyrosine-based switch motif (ITSM) at the cytoplasmic tail can inhibit the activation of T cells through the phosphorylation of src family kinases, recruiting SHP-1 and SHP-2 protein tyrosine phosphates (Zak et al., 2015). In contrast to CTLA-4, PD-1 is highly expressed on B cells and natural killer (NK) cells (Fanoni et al., 2011; Terme et al., 2011). PD-1 inhibits the activity of peripheral T cells and other autoimmune reactions when the body responds to inflammation, especially chronic inflammation (Keir et al., 2006; Topalian et al., 2016). In malignant tumors, PD-1 inhibits the activity of effector T cells, which is one of the main mechanisms by which tumor cells resist the immune system (Blank et al., 2004).

**FIGURE 2:**
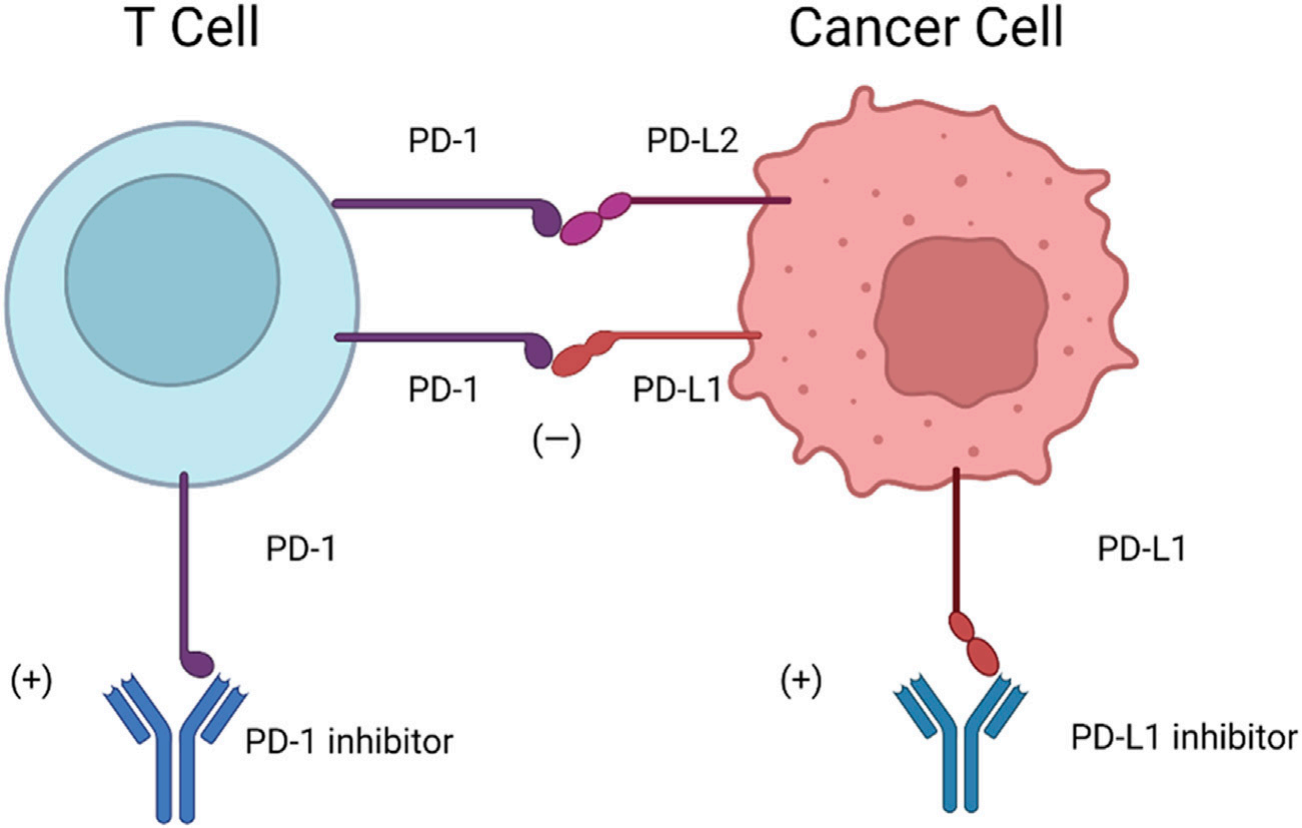
Mechanisms of PD-1-PD-L1/PD-L2. Cancer cells are highly capable of PD-L1 expression, which can inhibit the immune function of T cells in response to PD-L1 and PD-1 binding. Therefore, anti-PD-1 and anti-PD-L1 are considered promising strategies to stimulate “exhausted” T cells and eliminate tumors. PD-L2 is similar to PD-L1 in structure, has some sequence homology inhibits the function of T cell inhibition *via* PD-L2-PD-1.

Furthermore, PD-1 has two ligands: PD-L1 (also known as B7-H1 and CD274) and PD-L2 (also known as B7-DC and CD273) (Freeman et al., 2000; Latchman et al., 2001). PD-L1 is composed of IgV- and IgC-like extracellular domains, a transmembrane domain, and a short cytoplasmic tail. PD-L1 interacts with the extracellular domain of PD-1, changing its conformation (Lin et al., 2008). PD-L1 can be detected on the surface of hematopoietic cells, including DCs, macrophages, T cells, and B cells, and nonhematopoietic cells, including endothelial cells and keratinocytes. PD-L2 can be detected on macrophages and DCs (Liang et al., 2003). PD-L1 and PD-L2 partially share sequence homology, and both can bind to the coinhibitory receptor on T cells (Shin et al., 2005). The detailed mechanisms of PD-1-PD-L1/PD-L2 are shown in Figure 2.

## Immune checkpoint and osteosarcoma

Regarding primary and metastatic OS tumor sites, a previous study reported that PD-1 expression was increased in CD4^+^ and CD8^+^ T cells in peripheral blood (Zheng et al., 2015). IL-21 is required for the immune response of CD8^+^ T cells and is mainly secreted by circulating CD4^+^ T cells. Compared with those in healthy patients, circulating CD4^+^ T cells in OS patients have less capacity to secrete IL-21 due to the PD-1 and PD-L1 interactions of follicular helper T cells (Gao et al., 2017). In addition to T cells, the PD-1 and PD-L1 interaction also affects the cytotoxicity of NK cells. Blocking the PD-1/PD-L1 axis enhanced the ability of NK cells to lyse OS tumor cells by secreting granzyme B (Zhang et al., 2019). PD-L2 is also the ligand of the PD-1 receptor. PD-L2 protein was detected in primary OS and was increased in lung metastatic patients. According to the mechanism, the expression of PD-L2 is thought to enhance tumor growth and metastasis. *In vitro* experiments indicated that PD-L2 knockdown attenuated tumor growth and metastasis by inhibiting the RhoA-ROCK-LIMK2 pathway and autophagy (Ren et al., 2019).

In addition, many studies have reported a close relationship between the expression of PD-1/PD-L1 and the overall survival of OS patients. Koirala et al. (2016) found that PD-L1 expression at the OS tumor site was negatively related to 5-year event-free survival. However, after the immune cell composition was analyzed, the results indicated that PD-L1 expression was associated with the presence of T cells, DCs and NK cells. Hashimoto et al. (2020) reported that in OS, the tumor size was larger in PD-L1-negative cases than in PD-L1-positive cases, but the expression of PD-1 or PD-L1 did not alter the prognosis of patients who received chemotherapy and wide resection. Moreover, Yoshida et al. (2019) found that the expression of PD-L1 was related to early metastasis of OS, and PD-1 and PD-L1 expression were both negatively related to the prognosis of OS after the relationship between the expression levels and survival data of 62 patients were analyzed (Zheng et al., 2018). Consistently, a meta-analysis of eight studies and 413 OS patients was performed, and the pooled results showed that overexpression of PD-1 and PD-L1 led to an increased rate of metastasis and total mortality risk (Huang et al., 2018).

The binding of immune checkpoint receptors and their ligands inhibits T-cell function and other immune system components. Therefore, drugs targeting receptors and ligands are designed to prevent their binding, thus inhibiting the immune system. Researchers have conducted several animal experiments to evaluate the therapeutic effects of ICIs on OS. Yoshida et al. (2020) found that an anti-PD-1 antibody decreased OS tumor volume by decreasing Tregs in the tumor microenvironment (TME) and increasing tumor-infiltrating lymphocytes. Blockade of Blocking the PD-1/PD-L1 interaction decreased the tumor burden and extended survival in an OS mouse model by improving OS-reactive cytotoxic T lymphocytes (Lussier et al., 2015b). In addition, Dhupkar et al. (2018) found that an anti-PD-1 antibody decreased OS tumor cells in lung metastases by inducing apoptosis and inhibiting proliferation. Anti-PD-1 therapy increased the infiltration of NK cells and macrophages and the number of antitumor M1 macrophages and decreased M2 subsets. Interestingly, the PD-1 inhibitor nivolumab could inhibit lung metastasis of OS by increasing CD4^+^ and CD8^+^ lymphocytes and upregulating the cytotoxicity of CD8^+^ lymphocytes in the lung, but it was not effective for primary OS growth (Zheng et al., 2018).

## Clinical effects of immune checkpoint inhibitors on osteosarcoma

To date, several clinical trials have been carried out to examine the therapeutic effects of ICIs on OS (Table 1). The effects of PD-1 inhibitors, including pembrolizumab, nivolumab, and camrelizumab, the PD-L1 inhibitor atezolizumab, and the CTLA-4 inhibitor ipilimumab, on OS have been examined. Ipilimumab was used to treat 17 pediatric OS patients, and the results showed that it did not improve patient prognosis (Merchant et al., 2016). Pembrolizumab was used to treat 22 OS patients, and only one metastatic patient responded to this intervention (Tawbi et al., 2017). Clinicians treated 13 OS patients with nivolumab, and the results showed that the drug was well tolerated by children, but the therapeutic effect of this single agent was not observed (Davis et al., 2020). The PD-L1 inhibitor atezolizumab was used to treat 12 refractory or relapsed OS patients, and the results indicated that this drug was also tolerated by children but was ineffective (Geoerger et al., 2020). In addition, Le Cesne et al. (2019) reported that 17 advanced OS patients were treated with pembrolizumab and only one patient with a PD-L1-negative tumor had a partial response to the treatment.

**TABLE 1:** Current clinical trials of ICIs therapy on OS.

Agent	Target	Research stage	References
Pembrolizumab	PD-1 inhibitor	A completed phase 2 clinical trial	Boye et al. (2021)
Nivolumab	PD-1 inhibitor	A completed phase 1–2 clinical trial	Davis et al. (2020)
Atezolizumab	PD-L1 inhibitor	A completed phase 1–2 clinical trial	Geoerger et al. (2020)
Camrelizumab + Apatinib	PD-1 inhibitor + VEGFR inhibitor	A completed phase 2 clinical trial	Xie et al. (2020)
Nivolumab + Ipilimumab	PD-1 inhibitor + CTLA-4 inhibitor	A completed phase 2 clinical trial	D'Angelo et al. (2018)
Nivolumab + Azacitidine + surgery	PD-1 inhibitor + Cytosine nucleoside analog + Surgery	An ongoing phase 1–2 clinical trial	NCT03628209
Nivolumab + Sunitinib	PD-1 inhibitor + Tyrosine-kinase inhibitor	An ongoing phase 1–2 clinical trial	NCT03277924
Camrelizumab + Neoadjuvant chemotherapy	PD-1 inhibitor + Neoadjuvant chemotherapy	An ongoing phase 2 clinical trial	NCT04294511
SHR1210 + Apatinib	PD-1 inhibitor + VEGFR inhibitor	An ongoing phase 2 clinical trial	NCT03359018
Camrelizumab + MAPI + Apatinib	PD-1 inhibitor + Microbial alkaline protease inhibitor + VEGFR inhibitor	An ongoing phase 2 clinical trial	NCT04351308
Camrelizumab + Famitinib + Isosfamide	PD-1inhibitor + Tyrosine-kinase inhibitor + DNA synthesis inhibitor	An ongoing phase 2 clinical trial	NCT04044378
ZKAB001	PD-L1 inhibitor	Ongoing phase 1–2, and 3 clinical trials	NCT03676985, NCT04359550
Avelumab	PD-L1 inhibitor	An ongoing phase 2 clinical trial	NCT03006848
Pembrolizumab	PD-1 inhibitor	An ongoing phase 2 clinical trial	NCT03013127
Nivolumab + Ipilimumab	PD-1 inhibitor + CTLA-4 inhibitor	An ongoing phase 2 clinical trial	NCT02500797, NCT02982486
Durvalumab + Tremelimumab	PD-L1 inhibitor + CTLA-4 inhibitor	An ongoing phase 2 clinical trial	NCT02815995
MASCT-I+ anti-PD-1antibody + Apatinib	DC vaccine + PD-1 inhibitor + VEGFR inhibitor	An ongoing phase 1 clinical trial	NCT04074564

## Possible mechanisms of unsatisfactory outcomes

The prognosis of clinical trials for the treatment of OS is unfavorable. Researchers have determined several possible mechanisms to explain these frustrating outcomes. Immune checkpoint receptors are mainly expressed on T cells, and inhibitors can activate these cells. However, there are other kinds of inhibitory cells in the TME that facilitate the immune escape of OS cells. The TME of OS is a combination of various immune cells (DCs, macrophages, T cells, B cells, etc.), stromal cells (mesenchymal stem/stromal cells, fibroblasts), and surrounding mineralized extracellular matrix (ECM). The components and status of the TME determine the proliferation and metastasis of OS.

Macrophages are a type of mature monocyte circulating in the peripheral blood and are a subset of white blood cells derived from hematopoietic stem cells in the bone marrow (Shi and Pamer, 2011). Macrophages are able to assimilate debris, apoptotic cells and pathogens to maintain internal homeostasis (Murray and Wynn, 2011). According to their properties and functions, macrophages are classified into two categories: classically activated (M1) and alternatively activated (M2) macrophages (Shapouri-Moghaddam et al., 2018). In the early stage of OS formation, M1 macrophages play a dominant role in the TME, activating the immune system and inflammatory reactions to eliminate tumor cells. Related cytokines, including IL-1β, tumor necrosis factor-α (TNF-α), and IL-12, are locally released to increase the antitumor reaction (Hu et al., 2022). To avoid severe tissue and organ impairment caused by M1 macrophages, M2 macrophages, which are induced by IL-4/IL-10/IL-13, are designed to exert anti-inflammatory, profibrotic and proangiogenic effects (Wang et al., 2022). The switch from M1 macrophages to M2 macrophages is called polarization (Zhao et al., 2022). M2 macrophages in the TME are known as tumor-associated macrophages (TAMs) (Hasan et al., 2022). In the late stage of OS proliferation and metastasis, TAMs play a dominant role in the TME to support cancer cell growth and suppress immune reactions (Christofides et al., 2022). Based on these mechanisms, drugs targeting TAMs have been evaluated for their effects on OS patients. Drugs inhibiting TAM polarization and depleting or reprogramming TAMs are able to terminate immunosuppression in OS, such as mifamurtide, zoledronic acid, all-trans retinoic acid, and dihydroxycoumarin (Kimura and Sumiyoshi, 2015; Zhou et al., 2017; Lv et al., 2020; Barnes et al., 2022).

DCs are APCs that link innate and adaptive immunity (Mildner and Jung, 2014). In tumor immunity, DCs can capture malignant cell antigens and present them to T cells to start the expansion of tumor-specific T cells (Galluzzi et al., 2017). DCs are responsible for immunosurveillance; impairments in this mechanism are commonly observed in OS patients and lead to less effective therapeutic results and worse outcomes (Kroemer et al., 2013). DCs are distributed in nearly all tissues, and their functions depend on their population and maturation stages. Some subsets (such as CD103^+^ DCs) act as typical immune cells and present tumor antigens to T cells and other immune cells, such as NK and B cells. Some subsets (such as CD208^+^ DCs) predict an unfavorable prognosis (Bruni et al., 2020). Tumor cells can secrete several cytokines to inhibit the maturation of DCs, so researchers have attempted to treat malignant tumors using cancer vaccines targeting DCs (Michielsen et al., 2011). DCs can be isolated from peripheral blood mononuclear cells (PBMCs) and stimulated with tumor antigen *ex vivo*. Then, the cultured cells are injected back into patients. A DC cancer vaccine stimulated with MAGE-A1, MAGE-A3, and NY-ESO-1 was used to treat OS patients, and the results showed that the therapy was tolerated, but the effect was uncertain due to the limited number of recruited patients (Krishnadas et al., 2015).

Myeloid-derived suppressor cells (MDSCs) are another type of immunosuppressive immune cell in the TME (Lim et al., 2014). MDSCs are dramatically increased in the peripheral blood of OS patients (Shi et al., 2019). For these lung metastatic OS patients, the accumulation and activation of polymorphonuclear MDSCs can be detected at the metastasis (Ligon et al., 2021). MDSCs inhibit immune reactions through several methods. For example, MDSCs decrease the lymph node homing of CD4^+^ and CD8^+^ T cells to suppress the functions of T cells and NK cells (Najjar and Finke, 2013). MDSCs can also recruit and induce immunosuppressive Tregs (Dysthe and Parihar, 2020). In OS animal models, the level of IL-18 is positively correlated with the number of MDSCs in peripheral blood (Guan et al., 2017). The accumulation of MDSCs in the OS TME is also related to the activation of the PI3Kδ/γ and SDF-1/CXCR4 pathways (Jiang et al., 2019; Shi et al., 2019). All-trans retinoic acid can decrease the number of MDSCs in the OS TME (Long et al., 2016).

In addition to the immunosuppressive effects of the aforementioned immune cells, OS is a “cold tumor” compared with other kinds of tumors. PD-L1 is highly expressed on the surface of OS cells, suppressing the antitumor effects of immune cells. OS cells express nonimmunogenic properties due to the lack of specific antigens (Tsukahara et al., 2008; Bunnell et al., 2010). Genetic alterations were discovered in OS, which exhibits high copy number loss, especially in low immune infiltration conditions, revealing strong immunosuppression (Wu et al., 2020). Recent studies have reported that the expression of HER2 at low levels indicates the possibility of CAR-T therapy, which may turn a “cold tumor” into a “hot tumor” (Rainusso et al., 2012).

Compared with malignant tumors in the viscera or superficial soft tissue, OS is also closely related to the status of bone. The development of OS is correlated with osteolysis, and most OS patients were also diagnosed with fragility fractures. Osteoblasts, osteoclasts and osteocytes are involved in osteolysis. Osteoclasts are overactivated in the OS site, and the underlying mechanism is the binding of RANK and RANKL. However, the combination of chemotherapy and the RANKL inhibitor denosumab or biphosphates did not lead to a better prognosis (Cathomas et al., 2015; Piperno-Neumann et al., 2016). Therefore, treating OS by inhibiting osteolysis has not achieved a positive prognosis and needs further research. In addition, OS is not homogenous for all patients, and it can be classified into three subtypes: osteoblastic, chondroblastic, and fibroblastic (Gill and Gorlick, 2021). The expression of PD-L1 varies in different subtypes and in primary or metastatic tumors, indicating that OS is heterogenous (Lussier et al., 2015b). Therefore, the treatment of OS should be personalized according to the properties of the tumor.

## New therapeutic methods

To overcome the drug resistance of current ICI therapy, clinicians and researchers have been trying many new methods to enhance treatment efficacy.

### Precision therapy

ICI treatment is effective for some OS patients but ineffective for others. Finding the similarities between these ICI-responsive cases and selecting suitable patients before treatment could lead to a better prognosis. Starzer et al. (2021) recruited eight OS patients and analyzed the DNA methylation profile related to the immunology of tumor cells and the response to anti-PD-1 therapy. The most predominant differences in the DNA methylation profiles of responders and nonresponders were related to Rap1 signaling, adherens junctions, and focal adhesion. Patients with these methylation properties were more responsive to anti-PD-1 therapy.

### Synergistic application of immune checkpoint inhibitors

Single-agent use of ICIs seems unsatisfactory for treating OS, so synergistic use of multiple ICIs may lead to improved outcomes. Clinical trials have assessed the therapeutic effects of multiple ICIs. D'Angelo et al. (2018) reported combining the PD-1 inhibitor nivolumab and the CTLA-4 inhibitor ipilimumab to treat OS. Compared with the nivolumab-only group, patients who received the combination therapy had higher rates of response. In addition, animal studies evaluated the effect of synergistic treatment. Lussier et al. (2015a) alcombined anti-PD-1 therapy and anti-CTLA-4 therapy to treat mice with metastatic OS, and the results showed that the combination immunotherapy prevented the immune escape of tumor cells and led to complete control of metastatic OS.

### Combination with chemotherapy or radiotherapy

However, traditional therapies such as chemotherapy and radiotherapy exhibited unsatisfactory effects on OS. Recent studies showed that the combination of chemotherapy or radiotherapy with immunotherapy showed a better prognosis than a single application.

A small quantity of tumor-infiltrating immune cells decreased the efficacy of ICIs on OS. Deng et al. (2020) reported that neoadjuvant chemotherapy increased the numbers of CD3^+^, CD8^+^, and Ki67^+^ CD8^+^ T cells and PD-L1^+^ immune cells and decreased the numbers of MDSCs in the TME, converting OS from “cold” to “hot”.

In addition, although radiotherapy alone is insufficient for treating OS, several studies have reported that combining radiotherapy with ICIs could overcome the shortcomings of monotherapy. In 2018, Xia et al. (2018) first reported that in a mouse model, radiotherapy could enhance the efficacy of PD-1 inhibition on brain metastatic OS, increasing the number of CD8^+^ T cells in the TME. Katsuki et al. (2022) alfound a similar outcome of combination therapy in a mouse OS model. Callaghan et al. (2020) combined pembrolizumab with stereotactic body radiation to treat chondroblastic OS. Although the sample size was limited, the outcome proved that the combination therapy was well tolerated and that the prognosis was favorable.

Furthermore, carbon ion radiotherapy has been suggested to be more efficient for many kinds of malignant tumors than traditional radiotherapy (Kamada et al., 2015). Several recent studies showed that carbon ion radiotherapy could alleviate the drug resistance of PD-1 blockade therapy on OS. Permata et al. (2021) found that carbon ion radiotherapy upregulated the expression of PD-L1 on OS cells in a manner that was dependent on ATR kinase activity. Zhou et al. (2022) found that carbon ion radiotherapy triggered more immunogenic tumor cell death and increased the infiltration of CD4^+^ and CD8^+^ T cells, improving the efficacy of PD-1 blockade therapy for OS. Helm et al. (2021) treated OS mice with carbon ion radiotherapy combined with PD-1 and CTLA-4 inhibitors. ICIs or radiotherapy alone could not alleviate the progression of tumors, and the combination of the two treatments inhibited lung metastasis by increasing CD8^+^ T cells.

### Oncolytic viruses

Oncolytic viruses exert antitumor effects by directly lysing tumor cells. Mochizuki et al. (2021) designed the telomerase-specific oncolytic adenovirus OBP-502, which induces lytic tumor cell death by binding to integrins. Intratumoral injection of OBP-502 abrogated the restriction of PD-1 blockade on OS by enhancing tumor-infiltrating CD8^+^ T cells. In addition, Christie et al. (2021) genetically engineered myxoma virus to express TNF, and peripheral blood monocytes that were preloaded with the bioengineered virus increased the immune reaction and had an effective synergistic effect with anti-PD-1, anti-PD-L1, and anti-CTLA4 therapy.

### Others

In addition to the drugs and methods mentioned previously, many other drugs and interventions also have synergistic effects on OS when combined with ICIs. The growth and metastasis of tumors are accompanied by the formation of blood vessels. Vascular endothelial growth factor (VEGF) is an angiogenic factor that is essential for the formation of new blood vessels (Apte et al., 2019). Apatinib is a competitive inhibitor of VEGFR2 that is capable of inhibiting angiogenesis and carcinogenesis (Zhao et al., 2021). Xie et al. (2020) combined apatinib with the PD-1 inhibitor camrelizumab to treat advanced OS patients and followed up for 48 weeks. Combination therapy resulted in a better prognosis than the use of apatinib alone, especially for patients with PD-L1 overexpression or with lung metastasis.

In addition, MDSCs heavily infiltrate the TME of OS. Jiang et al. (2019) found that activation of the SDF-1/CXCR4 axis reduced MDSC apoptosis, upregulating the functions of Tregs. CXCR4 antagonists have synergistic effects with PD-1/PD-L1 inhibitors for treating OS. Sunitinib, a multitargeted receptor tyrosine kinase inhibitor, can activate the immune reaction by changing immune cell subsets. In OS, sunitinib reduced the population of Tregs and led to the DC-based cross-priming of IFN-γ-producing effector T cells. Sunitinib had a synergistic effect with nivolumab-mediated PD-1 blockade to treat OS (Ocadlikova et al., 2021). Secreted frizzled-related protein 2 (SFRP2) promotes the migration of tumor cells and tube formation in endothelial cells, which are associated with OS metastasis. An *in vitro* study showed that a monoclonal antibody against SFRP2 combined with a PD-1 antibody synergistically inhibited the metastasis of OS (Nasarre et al., 2021). Recruitment of MDSCs to the TME inhibits the effect of anti-PD1 therapy. Shi et al. (2019) reported that SNA, a specific inhibitor of PI3Kδ/γ, could inhibit the function of MDSCs and had a synergistic therapeutic effect with anti-PD1 therapy on OS tumor-bearing mice.

## Conclusion

OS is the most common primary musculoskeletal malignant tumor. Traditional therapies, such as surgery, chemotherapy, and radiotherapy, result in barely satisfactory prognoses for OS, and their side effects are serious. Immunotherapy is used to activate the inhibited immune system of OS patients, and ICIs have attracted the attention of researchers, clinicians, and patients. PD-1, PD-L1, and CTLA-4 inhibitors are representative ICIs. Their therapeutic effects on OS are not as good as those on hematopoietic tumors. We elucidated the possible underlying mechanisms and summarized the current research on alleviating ICI drug resistance in OS. By further determining the underlying mechanisms and developing drug design and drug administration technology, the therapeutic effects of ICIs on OS will be dramatically improved in the future.
